# Is type 2 diabetes an adiposity-based metabolic disease? From the origin of insulin resistance to the concept of dysfunctional adipose tissue

**DOI:** 10.1007/s40519-021-01109-4

**Published:** 2021-02-08

**Authors:** Paolo Sbraccia, Monica D’Adamo, Valeria Guglielmi

**Affiliations:** 1 Dipartimento di Medicina dei Sistemi, Università di Roma “Tor Vergata”, Via Montpellier, 1, 00133 Rome, Italy; 2 Unit of Internal Medicine, Obesity Center, Policlinico Tor Vergata, Rome, Italy

**Keywords:** Type 2 diabetes, Obesity, Insulin resistance, Fat mass, Adipose tissue

## Abstract

In the last decades of the past century, a remarkable amount of research efforts, money and hopes was generated to unveil the basis of insulin resistance that was believed to be the primary etiological factor in the development of type 2 diabetes. From the Reaven’s insulin resistance syndrome to the DeFronzo’s triumvirate (skeletal muscle, liver and beta-cell) and to Kahn’s discovery (among many others) of insulin receptor downregulation and autophosphorylation, an enthusiastic age of metabolic in vivo and in vitro research took place, making the promise of a resolutory ending. However, from many published data (those of insulin receptoropathies and lipodystrophies, the genome-wide association studies results, the data on reversibility of type 2 diabetes after bariatric surgery or very-low-calorie diets, and many others) it appears that insulin resistance is not a primary defect but it develops secondarily to increased fat mass. In particular, it develops from a mismatch between the surplus caloric intake and the storage capacity of adipose tissue. On this basis, we propose to change the today’s definition of type 2 diabetes in adiposity-based diabetes.

***Level of Evidence*** as a narrative review a vast array of studies have been included in the analysis, ranging from properly designed randomized controlled trials to case studies; however, the overall conclusion may be regarded as level IV.

## Introduction

No area in endocrinology and few areas in all biomedicine research fields have had such an extraordinary scientific explosion as insulin resistance. From the few initial reports of diabetic patients requiring very high doses of insulin [1, 2], the real cornerstone was the development of radioimmunoassay (RIA) of peptide hormones by Solomon Berson and Rosalyn Yalow [3]. For this great advancement, that marked the modern era of hormone research, R. Yalow was awarded the Nobel Prize in Physiology or Medicine in 1977. Consequently, for the first time, it was proven that in type 2 diabetes insulin was inefficient rather than deficient [4]. This change in paradigm, at those times, revolutionized the way the etiology and pathophysiology of type 2 diabetes was viewed.

The somehow turbulent race to uncover the mechanisms underlying insulin resistance, both in vivo and in vitro, tended to progressively obscure the role of an increase in fat mass due to a positive energy balance.

Therefore, the path that from Hippocrates and Morgagni to Vague and others, led to the dominant thinking that adult diabetes was the result of obesity was interrupted.

Hippocrates wrote: “It is very injurious to health to take in more food than the constitution will bear, when, at the same time one uses no exercise to carry off this excess” [5]; Joannes Baptista Morgagni, some 270 years ago, recognized the association between visceral obesity, hypertension, hyperuricemia, atherosclerosis, and obstructive sleep apnoea syndrome [6]. Finally, Jean Vague, in 1947, pointed again to the central (android) obesity phenotype and its association with diabetes, atherosclerosis, gout, and uric-acid calculus disease [7].

However, at those times and in part even nowadays, there was a sort of stigma in considering obesity more than an aesthetic problem due to gluttony and sedentariness, and the endocrinology world welcomed enthusiastically the theory that insulin resistance could have been the primary pathogenic defect to trigger type 2 diabetes or, as called those days, “maturity-onset diabetes”. As a matter of fact, way back in 1933, at the annual meeting of the American Physicians and Surgeons in Washington D.C., a renowned scientist introduced his talk by saying: “To be an endocrinologist among the practising profession today means too often to be primarily concerned with making fat ladies thin” [8].

Therefore, in particular until the discovery of leptin in 1992 [9], a milestone for the upsurge interest in adipose tissue, a remarkable amount of research efforts, money and hopes was generated to unveil the basis of insulin resistance. In vivo and in vitro studies on insulin resistance fed each other, floating all the possible scientific spaces of the metabolic arena, thus excluding obesity in the pathogenesis of type 2 diabetes [13–23, 36–40]. With the subsequent growing interest in adipose tissue, the role of increased adiposity progressively regained scientific interest, at least as a risk factor for the development of insulin resistance [10, 11].

*Historia magistra vitae* is a Latin expression, used by Cicero in his De Oratore [12], that means “history (is the) teacher of life” and that conveys the idea that the study of the past should serve as a lesson to the future or, at least, to the present. In this light, we firmly believe that science should not only transform theories in scientific truth when they are rigorously verified but also to dismiss those theories that, even after many decades, become progressively weaker and blurred.

This narrative review, therefore, aims to recapitulate, within an historical perspective encompassing approximately 70 years, the major scientific milestones and hypothesis on the etiopathogenesis of type 2 diabetes, with a specific focus on the putative overestimation of the role of insulin resistance and an underestimation of the role of chronic positive energy balance in the etiopathogenesis of type 2 diabetes.

## Insulin resistance: setting the scene

The breakthrough discovery that plasma insulin concentrations were higher in maturity-onset diabetes compared to non-diabetic individuals after an oral glucose challenge [4], opened a long-lasting season of intense research into both the metabolic and molecular mechanisms underlying this phenomenon. Based on their results Berson and Yalow concluded: “*that the tissues of the maturity-onset diabetic do not respond to his insulin as well as the tissues of the nondiabetic subject respond to his insulin*”: the insulin resistance era was born.

Although many outstanding scientists contributed significantly to the advancement of this new fascinating field in the years that followed [13–17], three of them may well be considered as fathers of insulin resistance: Gerald Reaven, Ralph DeFronzo and, last but certainly not least, Ronald Kahn; all three were recipients of the prestigious Banting Medal for scientific achievement conferred by the American Diabetes Association (ADA) [18–20].

Their many scientific contributions mirrored the main stream thinking on this topic of the last decades of the twentieth century.

### Gerald Reaven, the primacy of insulin resistance and the Syndrome X

He pioneered the concept that insulin resistance could be a primary defect leading to compensatory hyperinsulinemia that on one hand may lead to type 2 diabetes when the beta-cells compensatory response declines [21] and, on the other hand, to the “Insulin resistance syndrome” or Syndrome X that increases the cardiovascular risk [18, 22]. In a seminal paper, he demonstrated that 25% of healthy individuals have a degree of insulin-stimulated glucose uptake that is superimposable to that of type 2 diabetic patients [23]; in other words, he proposed that insulin resistance is prevalent in the normal population and it is not a consequence of the metabolic derangements of uncontrolled diabetes. Those normal subjects primarily insulin resistant are, therefore, at risk of developing type 2 diabetes. In addition, these data demonstrated that insulin resistance precedes the onset of the disease, suggesting that it may be an initial abnormality. One criticism that might be raised to this line of reasoning is that the quartile with the worse insulin sensitivity was composed mainly by males and had a mean body mass index (BMI) that was in part in the overweight range, as typical for North American individuals. Looking at these data today, it is reasonable to assume that the insulin resistance in those individuals defined “healthy”, was secondary to the typical combination of chronic caloric surplus and sedentary lifestyle.

As far as the Syndrome X is concerned, it underwent various reconsideration, reclassifications and critical appraisal [24–28]. We believe that in spite of all the limitations of the various definitions, the one proposed by the International Diabetes Federation [26], i.e., the presence of either obesity or increased waist circumference, is supported by strong evidences linking a state of chronic positive energy balance to the development of all the component of the Metabolic Syndrome (alias Sn. X) [29–31]. Insulin resistance, therefore, is not a primary defect but it develops secondarily to increased fat mass [32–35].

### Ralph DeFronzo, the glucose clamp: from the triumvirate to the “hateful eight”.

He had the invaluable merit of having developed, together with Jordan Tobin and Reubin Andres, the gold standard for measuring in vivo insulin sensitivity: the euglycemic hyperinsulinemic clamp [36]. This technique, coupled with the use of isotopic tracers, enable also to investigate key metabolic parameters such as hepatic glucose output and insulin secretion. By systematically applying these techniques, DeFronzo, with many other investigators worldwide who had the privilege of being initiated to the holy grail of glucose clamp, drew a sophisticated map of the metabolic fluxes controlled by insulin in both physiological and pathological conditions. These exceptional scientific achievements were brilliantly synthesized during both the ADA 1987 Lilly Lecture [37] and the ADA 2008 Banting Lecture [19]. In 1987, DeFronzo applied a metaphor taken from ancient Rome, the triumvirate (i.e., a board of three officials), to explain the pathogenesis of Type 2 diabetes: insulin resistance in skeletal muscle and liver, and beta-cell failure [37]. In the following 20 years, this view grew to include also fat cell (accelerated lipolysis), gastrointestinal tract (incretin deficiency/resistance), alpha-cell (hyperglucagonemia), kidney (increased glucose reabsorption), and brain (insulin resistance): the ominous octet [19].

From the seminal work of DeFronzo’s group [19, 37], as well as of other investigators [38, 39], the leading idea was that skeletal muscle insulin resistance was the major contributing factor to postprandial hyperglycemia in type 2 diabetes, or as defined in those years “non-insulin-dependent diabetes mellitus” (NIDDM), and in part also in the fasting state when liver represents a major site of insulin resistance thus increasing its glucose output. It is noteworthy that, on the basis of the aforementioned results, adipose tissue glucose uptake was assumed to represent around 1% of total glucose disposal [38, 39]; therefore, fat mass was considered an inert compartment as far as glucose metabolism is concerned. Thus obesity, that contribute to insulin resistance through the increased plasma concentrations of free fatty acids and the consequent enhanced lipid oxidation, was viewed as a special condition somehow distinct from the pathogenetic picture of NIDDM [40].

The conclusion of most scientists at that time was that since NIDDM is a complex disease in which is difficult to identify the *primum movens* in spite of intense investigations, it must come into play a genetic susceptibility to insulin resistance. In this regard, DeFronzo in 2008 wrote: “Individuals destined to develop type 2 diabetes inherit a set of genes from their parents that make their tissues resistant to insulin … As long as the beta-cells are able to augment their secretion of insulin sufficiently to offset the insulin resistance, glucose tolerance remains normal. However, with time the beta-cells begin to fail and initially the postprandial plasma glucose levels and subsequently the fasting plasma glucose concentration begin to rise, leading to the onset of overt diabetes” [19].

In the following pages, we will specifically challenge these conclusions since neither data exist that show genes directly inducing insulin resistance in type 2 diabetes nor data that support beta-cells failure secondary to chronic insulin resistance per se in the absence of positive energy balance.

### Ronald Kahn, the insulin receptor down-regulation and autophosphorylation, tissue-specific insulin receptor, IRS1 and IRS2 knockout mice

He made two groundbreaking discoveries (insulin receptor downregulation and autophosphorylation) and many seminal observations, especially those derived from the tissue-specific insulin receptor, IRS1 and IRS2 knockout mice experiments. For these outstanding achievements, he received many prestigious awards; in particular, the Banting Medal for scientific achievement from the American Diabetes Association in 1993 [20] and the George M. Kober Medal from the Association of American Physicians in 2019 [41]. In our opinion, he would have also deserved the Nobel Prize, if it came out that insulin resistance had indeed an etiologic role in the development of type 2 diabetes.

Ronald Kahn began his scientific career in the early seventies at the *National Institutes of Health* (NIH) under the mentorship of Jesse Roth; in the following years, Roth’s lab opened up the all field of receptor biology and insulin signaling; the group was composed of extremely talented scientists that joined NIH also thanks to the Vietnam War. Few alternatives to military service was the Clinical Associate Training Program at the NIH; since only a small percentage of applicants were accepted, this elite program launched an unprecedented number of remarkable scientific careers that would revolutionize medicine at the end of the twentieth century [42]. Therefore, the basic counterpart of in vivo insulin metabolism was particularly powered also by external geopolitical factors.

Later on, Kahn’s group applied the Cre-loxP technique [43] to develop a vast array of tissue-specific knockout models of insulin resistance [44]. In particular, the muscle-specific insulin receptor knockout (MIRKO) mice provided unexpected results. This mouse model showed no alteration in glucose homeostasis, although it displayed elevated serum triglycerides, free fatty acids and increased visceral fat mass [44]. For the first time, it was recognized that insulin receptor signalling in muscle is not necessary to maintain post-prandial glucose disposal in mice.

Overall, these three giants in the field of insulin resistance, contributed significantly in spreading the concept that insulin resistance is, or at least may be, the initial defect in type 2 diabetes.

Most of the papers published at that time began with a sentence like the following: “Insulin resistance is a characteristic feature of patients with type 2 diabetes mellitus that precedes the onset of the disease. The presence of insulin resistance leads to increased beta-cell insulin secretion with compensatory hyperinsulinemia. When the beta-cell compensatory response declines type 2 diabetes ensues”.

As the molecular mechanisms of insulin signaling were progressively unveiled, all the newly identified molecules were investigated. In many cases skeletal muscle biopsies of patients with overt type 2 diabetes or glucose intolerance were analyzed to measure gene and/or protein expression and function of insulin receptor tyrosine kinase activity, inhibitors of tyrosine kinase activity, insulin receptor isoforms (IR-A and IR-B), insulin receptor substrate (IRS)-1 and IRS-2, phosphatidylinositol 3-kinase (PI3K)-AKT, glucose transporter-4 (GLUT-4), just to cite the most important ones [45–51]. These same molecules, and many others, served also as candidate genes to search for variants able to explain the skeletal muscle insulin resistance [52]. It turned out that all the proteins downstream the insulin receptors had a reduction in number and/or of function, but no variants were identified to upgrade them to the role of primary defect responsible for insulin resistance. These are, therefore, secondary defects and may be viewed as molecular effectors of chronic caloric energy balance and/or sedentariness.

As a corollary, we would like to envisage that Ronald Kahn is a case of missing Nobel Prize. In the late sixties, Ronald Kahn took the place of Robert Lefkowitz in the lab of Jesse Roth at NIH. Both were put on a project that aimed at developing a radioligand binding technique to study the adrenal adrenocorticotropic hormone (ACTH) receptor. But it did not work well for both. However, as it always happens for thoroughbreds, they both found their very successful ways. Ronald Kahn began to work with the insulin receptor whereas Robert Lefkowitz with the adrenergic receptors and more in general with G-protein-coupled receptors. They both made unbelievable scientific achievements, becoming soon worldwide recognized absolute leaders in their respective fields. They both received endless number of prestigious awards and medals. However, Robert Lefkowitz was awarded the 2012 Nobel Prize in Chemistry and Ronald Kahn not (yet?). The prize motivation was that approximately half of all medications used today make use of G-protein-coupled receptors.

We believe that if insulin action would have kept the ptolemaic promise of the golden age of being at the center of the metabolic universe, Ronald Kahn would have been awarded too.

## Lessons from the human syndromes of insulin resistance

The syndromes of extreme insulin resistance and the lipodystrophies have taught us two major lessons: 1. the clinical manifestations of true primary insulin resistance; 2. how secondary insulin resistance may arise from dysfunctional adipose tissue and lipotoxicity.

The following considerations will be made exclusively with the aim to elucidate these two aspects.

### Primary extreme insulin resistance syndromes: mutations in the insulin receptor gene.

Leprechaunism, type A insulin resistance and Rabson-Mandenhall are the three rare syndromes characterized by mutations in the insulin receptor gene [53]. Leprechaunism bears mutations that impair so severely the insulin receptor function that patients usually die in the first year of life. On the contrary, type A insulin resistance, characterized clinically by *acanthosis nigricans* and hyperandrogenism, is compatible with a life close to normal. The Rabson–Mendenhall syndrome is defined by the presence of several additive clinical features that include abnormalities of teeth and nails, and pineal hyperplasia; from a clinical point of view, it appears to be intermediate in severity between leprechaunism and type A insulin resistance. Simeon Taylor at the NIH made the first genetic characterization of these patients identifying mutations that affect either the binding affinity to insulin or the activation of the tyrosine kinase in the intracellular portion of the receptor. He pioneered this field under the initial mentorship of Jesse Roth and Phil Gorden [54, 55].

These patients are characterized by marked hyperinsulinemia needed to overcome the genetically dysfunctional insulin receptor. The patients with homozygous mutations are the more resistant and may require several thousand units of insulin per day. However, some of the milder forms (mainly type A insulin resistance) may have close to normal glucose tolerance in spite of congenital markedly elevated plasma insulin concentrations. What happens to their beta-cells after many years of insulin hypersecretion? An answer to this question came from some of the patients studied after 30 years from the initial diagnosis of either type A insulin resistance (n. 8) or with Rabson-Mandenhall syndrome (n. 3) [56]. In particular, two of them with Type A insulin resistance and female were normoglycemic at diagnosis 30 years earlier when they were in their prebubertal—pubertal age. Both had plasma insulin concentration > 250 µU/ml in the fasting state (normal values < 20) and around 1000–2000 µU/ml postprandially (normal values < 100). One of the two, 26 years later continued to be normoglycemic and normotolerant with plasma insulin concentrations that continued to range between one hundred and one thousand in the fasting and postprandial state, respectively. The other patient developed only mild impaired glucose tolerance in spite of extremely elevated plasma insulin concentrations. Nine out of the eleven patients were and remained normal weight and were normotrygliceridemic.

Therefore, these data, although obtained in a very limited number of patients, fulfill the proof of concept that primary insulin resistance does not lead necessarily to decline in beta-cell function. In addition, from further studies, it has been confirmed that patients with insulin receptoropathy are normal-weight and protected from developing liver steatosis and the features of metabolic syndrome [57]. As far as body weight is concerned, two robust longitudinal studies carried out either within the San Antonio Heart Study or in the Pima Indians of Arizona, have clearly shown that insulin resistance protects from weight gain [58, 59].

### Secondary severe insulin resistance syndromes: consequences of pathological adipose tissue deficiency

During the receptor-centric years it was thought that also the lipoatrophic diabetes (as lipodystrophies were defined at that time) resulted from mutations in the insulin receptor gene [60]. Later on, it became clear that lipodystrophies are caused by a primary selective generalized or partial loss of white adipose tissue [61, 62]. They are a heterogenous group of either genetic or acquired syndromes that, beyond their complex pathogenesis, are all characterized by the absent/limited adipose tissue storage capacity with consequent ectopic deposition of free fatty acids in skeletal muscle, liver, pancreas, and the visceral adipose tissue (in the partial forms characterized by selective subcutaneous adipose tissue loss) [63]. The chronic lipotoxic environment leads to diabetes mellitus, in the most severe forms recurrent episodes of acute pancreatitis from extreme hypertriglyceridemia, cirrhosis resulting from long-standing hepatic steatosis, and atherosclerotic vascular disease [61].

It is remarkable that the physiopathological picture resulting from the reduction/absence of adipose tissue is superimposable to the typical metabolic syndrome of the overweight/obese individuals: atherogenic dyslipidemia (hypertriglyceridemia, low HDL cholesterol), impaired glucose tolerance/overt diabetes, non-alcoholic fatty liver disease (NAFLD).

Therefore, unravelling the aetiopathogenesis of lipodystrophies provided the clear evidence that metabolic syndrome results from a mismatch between the surplus caloric intake and the storage capacity of adipose tissue. White adipose tissue adapts and expands in response to surplus energy through adipocyte hypertrophy and proliferation of precursor cells in combination with vascular and extracellular matrix remodelling. However, in the context of chronic positive energy balance, adipocytes become dysfunctional due to fibro-inflammation processes [64] and the excess energy is redirected toward liver, pancreas [65] and skeletal muscle [66] thus contributing to increased risk of type 2 diabetes and cardiovascular diseases [67]. Furthermore, the adiposopathy (sick fat) concept explains why not all persons with overweight or obesity develop type 2 diabetes: it is not the amount of fat but its quality that is metabolically detrimental.

In summary, these two human models contributed significantly in highlighting the role of primary insulin resistance versus the secondary metabolic derangements due to inadequate energy storage capacity. Insulin resistance per se, as inferred from the insulin receptoropathies, is devoid of any effect on fat mass, triglycerides and HDL cholesterol; in addition, NAFLD is absent and it is, at present, unknown if it exerts any effect on the vasculature (Table 1).

**Table 1:** Anthropometric, metabolic and hormonal differences between obesity, lipodystrophies and insulin receptoropathy

Characteristics	Obesity $\uparrow$ (with Metabolic Syndrome)	Lipodystrophies	Insulin receptoropathy
BMI	$\uparrow$	$\downarrow$	$\leftrightarrow$
Body fat %	$\uparrow$	$\downarrow \downarrow \downarrow$	$\leftrightarrow$
Waist (cm)	$\uparrow$	$\leftrightarrow \downarrow$	$\leftrightarrow$
Hip (cm)	Relative $$\downarrow$$	$$\downarrow$$$$\downarrow$$	$$\leftrightarrow$$
Waist/hip ratio	$\uparrow \uparrow$	$\uparrow \uparrow$	$\leftrightarrow$
Insulin resistance	$\uparrow$	$\uparrow \uparrow$	$\uparrow \uparrow \uparrow$
Triglycerides	$\uparrow$	$\uparrow \uparrow$	$\leftrightarrow$
HDL cholesterol	$\downarrow$	$\downarrow$	$\leftrightarrow$
Leptin	$$\uparrow \uparrow$$	$$\downarrow$$$$\downarrow$$$$\downarrow$$/$$\downarrow$$	$$\leftrightarrow$$
NAFLD	$\uparrow$	$\uparrow \uparrow$	Absent
PCOS	$\uparrow$	$\uparrow \uparrow$	$\uparrow \uparrow$
Atherosclerosis	$\uparrow$	$\uparrow \uparrow$	Unknown

Modified from [63]

*NAFLD* non-alcoholic fatty liver disease, *PCOS* polycystic ovary syndrome

## How the Pima Indians challenged the primacy of insulin resistance in the development of type 2 diabetes

**Fig. 1:**
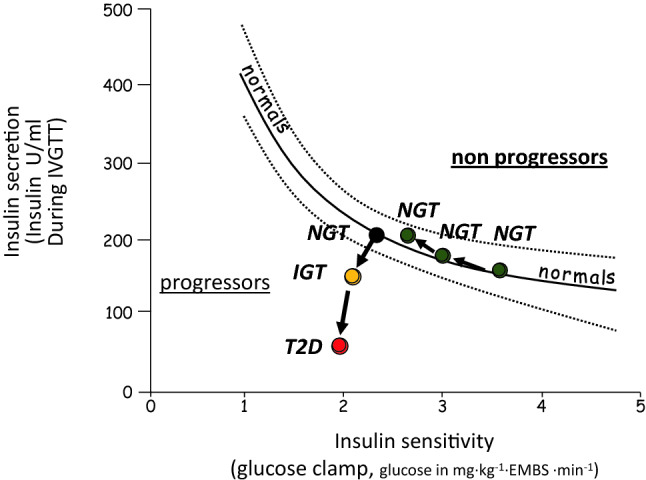
Insulin secretion and sensitivity changes and progression toward type 2 diabetes in Pima Indians. *NGT* normal glucose tolerance, *IGT* impaired glucose tolerance, *T2D* type 2 diabetes, *EMBS* estimated metabolic body size. Modified from [68]

Although beta-cell failure was always included in the definition of type 2 diabetes, the disease has been traditionally understood as a metabolic disorder initiated by insulin resistance. This view was necessarily reconsidered when, in 1999, data were published on the temporal sequence with which these metabolic abnormalities develop relative to one another during the different stages of the disease. The authors measured insulin action, insulin secretion, and hepatic glucose production longitudinally in Pima Indians, in whom glucose tolerance deteriorated from normal to impaired to diabetic over several years [68]. They demonstrated that as long as the insulin secretory response increases to compensate for the worsening of insulin sensitivity, subjects remain normoglycemic. The progression toward impaired glucose tolerance and frank diabetes is totally accounted for by impaired insulin secretion (Fig. 1).

## Did genetic help in solving the apparent conundrum?

At the end of the nineties, the leading hypothesis was that type 2 diabetes represented a definite and complex nosographic entity caused by the interplay of insulin resistance and beta cell failure. Obesity and environmental factors had additive deleterious, though aetiologically marginal, effects. As high-density single nucleotide polymorphism arrays (SNPs) became available for launching genome-wide association scans in large case–control cohorts, optimism pervaded the field. However, results did not go in the direction expected. In synthesis, 1. the larger genome-wide association studies (GWAs) identified loci with an effect size that barely goes beyond odd ratios of one (with very few exceptions) and, therefore, that confer a very small risk [69–72]; 2. most of the risk SNPs affect beta-cell function favouring a beta-cell-centric view on the genetics of type 2 diabetes [73].

Moreover, in an attempt to look for loci associated with insulin resistance phenotypes, a group headed by Stephen O’Rahilly and Robert Scott from the University of Cambridge, using an integrative genomic approach, identified 53 genomic regions associated with a limited capacity to store fat in a healthy way [74]. Therefore, also under a genetic point of view, insulin resistance appears a secondary hallmark of a dysfunctional adipose tissue.

**Fig. 2:**
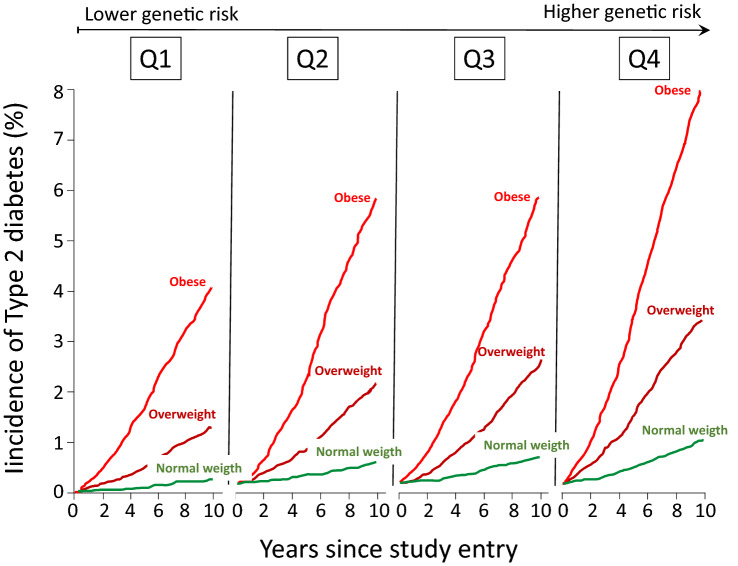
Risk of type 2 diabetes in relation of quartiles of genetic risk score and weight status. Modified from [75]

Of particular interest were those studies that investigated the genetic-lifestyle interaction to disentangle their relative contribution to confer risk of developing type 2 diabetes. To this end and taking advantage of the large EPIC InterAct Case-Cohort Study (12,403 incident type 2 diabetes cases from a cohort of 340,234 European participants with 3.99 million person-years of follow-up) Langenberg et al. were able to show that modifiable factors, particularly obesity, outweighed genetic risk scores in conferring absolute risk of developing type 2 diabetes. Obese individuals in the lowest genetic risk quartile were much more likely to develop type 2 diabetes than normal weight individuals in the highest genetic risk quartile (Fig. 2), indicating that if only genetics had been used for risk stratification, the individuals at highest risk would not have been targeted for intervention [75]. Similarly, in a case-cohort study carried out in Denmark, obesity and unfavourable lifestyle were associated with higher risk for incident type 2 diabetes regardless of genetic predisposition [76].

Therefore, the various genetic loci that were identified thanks to very large GWAs, mainly involved in insulin secretion rather than in insulin sensitivity, confer a modest susceptibility risk to develop type 2 diabetes, whereas obesity represents the strongest predictor.

## From bariatric surgery DiRECTly toward type 2 diabetes reversibility

**Fig. 3:**
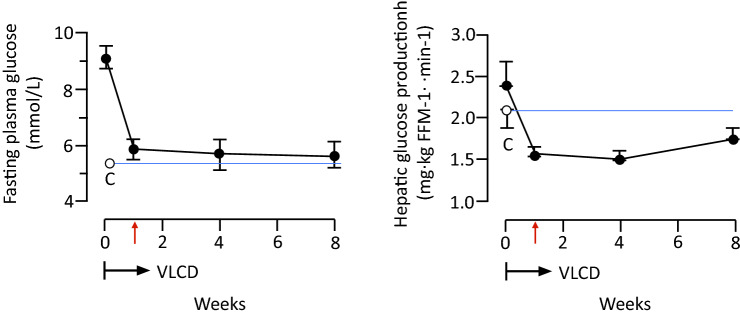
Effect of 8 weeks of very-low-calorie diet (VLCD) on plasma glucose (left panel) and basal hepatic glucose production (right panel). White circles and blue lines (C) indicate the mean for the weight-matched non-diabetic control group. Modified from [81]

Surgeons learned, over the years, that type 2 diabetes is a complex heterogeneous disease mainly driven by insulin resistance with a genetic predisposition, inevitably progressive despite glucose-lowering treatment, with 50% of individuals requiring insulin therapy within 10 years [77]. Therefore, it is not surprising that when they documented a complete withdrawal of all antihyperglycemic medications, including high dosage insulin, few weeks after a bariatric surgery intervention and well ahead of any significant weight loss, they claimed that something intrinsically to the intervention cured the disease [78, 79]. From this initial observation, a huge florilegium of hypothesis was put forward trying to explain the antidiabetic mechanisms of bariatric surgery [80]. However, it was completely overlooked a remarkable metabolic change that occurs after surgery, in particular during the first weeks: a striking decrease in caloric intake. This effect translates in profound changes in metabolic fluxes and intracellular handling of nutrients, in particular fat metabolites. To test the hypothesis that by reducing caloric intake, insulin resistance and beta-cell failure can be reversed, Roy Taylor and his group measured basal hepatic glucose output, hepatic and peripheral insulin sensitivity, beta cell function, and pancreas and liver triacylglycerol content in a small group of patients with type 2 diabetes before and during a very-low-calorie diet (VLCD) [81]. Quite surprisingly, within 7 days, liver fat decreased becoming similar to that of the control group, and hepatic insulin sensitivity normalized. Plasma glucose normalized by day 7 of the diet (Fig. 3). It is worth noting that, in spite of these striking results, peripheral insulin-mediated glucose disposal, as measured by glucose clamp, remained unchanged during the 8 weeks of the study. This seminal observation paved the way to further studies [82, 83] and led to a remarkable paradigm shift: type 2 diabetes is a reversible disease.

**Fig. 4:**
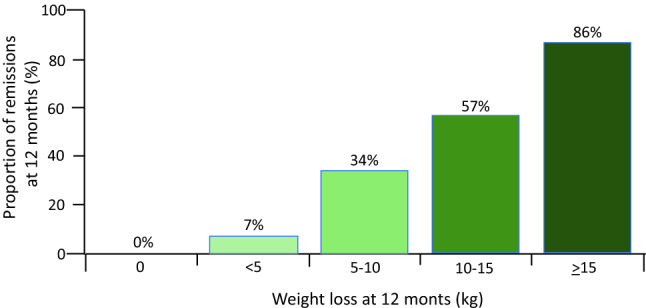
Remission of diabetes in relation to weight loss at 12 months of the DiRECT trial. Modified from [84]

To confirm these results in a larger and more robust setting, 298 type 2 diabetic patients were randomized into the Diabetes Remission Clinical Trial (DiRECT) to receive either a weight management programme (that included a low energy formula diet of 825–853 kcal/day for 3 months) or a best-practice care by guidelines as control. After 1 year almost half of participants in the intervention arm achieved remission to a non-diabetic state and off antidiabetic drugs [84]. Moreover, by stratifying the remissions rate by the amount of weight loss at 12 months, it was shown that the remission rate in those who lost ≥ 15 kg was close to 90% (Fig. 4). Therefore, beyond the proof of concept, remission of type 2 diabetes may represent also a practical target. This result was further corroborated in two recent studies that compared the metabolic effects of a VLCD versus Roux-en-Y gastric bypass in type 2 diabetic patients; the metabolic improvements were identical in the two group, ruling out any mechanism intrinsic to the surgical operation and confirming the reversibility of type 2 diabetes [85, 86].

These data strengthen the concept that type 2 diabetes is caused by a chronic condition of positive energy balance with the consequent deposition of caloric surplus in liver and pancreas when adipocytes become dysfunctional. As long as the beta-cells are not irreversibly damaged by long-lasting disease [87], the induction of a negative energy balance is able to reverse type 2 diabetes.

## Type 2 diabetes and the “metabolically obese” normal weight individuals: the role of fat distribution

Abdominal obesity is more closely associated with the risk of developing type 2 diabetes and several studies have suggested that waist circumference or the waist-to-hip ratio may be better predictors than BMI [88–90]. BMI has two major limitations: it is not a measure of fat mass, and it does not convey any information on fat distribution and regional fat depots, and for any given BMI, percentage body fat [91, 92] and fat distribution can vary substantially. This led to the identification, in terms of risk of developing the metabolic syndrome and type 2 diabetes, of two opposite phenotypes: the “metabolically healthy” obese individuals and the “metabolically obese” normal weight individuals [93, 94]. This latter phenotype is characterized by BMI < 25 but increased percentage body fat and increased waist circumference [95]. Therefore, not only the majority of individuals with type 2 diabetes are overweight or obese, but also the majority of type 2 diabetic patients with BMI < 25 have increased fat depots [95]. Moreover, if we consider that the majority of type 2 diabetic patients who are truly lean have positive islet cell antibodies and/or insulin autoantibodies belonging, therefore, to the LADA group of patients [96], the remaining *bona fide* type 2 diabetic patients with BMI < 25 and normal body composition with no secondary etiologies (pancreatopathies, endocrinopathies, etc.) represents a tiny portion that need further investigations.

## Is it time to revise the classification of diabetes?

WHO classification of diabetes moved from an age-related definition (e.g., maturity-onset) in 1965, to a therapeutic-related definition (e.g., non-insulin dependent diabetes mellitus – NIDDM) in 1980 and finally, in 1999, introduced the actual categorical division in type 1 and type 2 diabetes [97]. While the American Diabetes Association states that: “Type 2 diabetes is due to a progressive loss of b-cell insulin secretion frequently on the background of insulin resistance” [98], WHO at least acknowledges that it is: “commonly associated with overweight and obesity” [97].

There are some genetic and many secondary causes of diabetes that resemble type 2 diabetes, like monogenic defects of β-cell function (MODY), latent autoimmune diabetes of the adult (LADA), diseases of the exocrine pancreas, endocrinopathies, drug- or chemical-induced forms, hemochromatosis, etc., but these represents a tiny portion in comparison of the huge pandemic numbers of type 2 diabetic patients that are overweight, obese or that, even if their BMI is below 25, have increased fat depots as a result of a slight but chronic positive energy balance. In this setting the concept of personal fat threshold proposed by Taylor and Holman [99], may be applied. They hypothesize that each individual has a personal fat threshold, independent of BMI, which, if exceeded, increases the likelihood to develop type 2 diabetes. Subsequent weight loss to take the individual below their level of susceptibility should allow return to normal glucose control. The concept of personal fat threshold may be of practical benefit in explaining the onset of diabetes in the non-obese people with type 2 diabetes. For all the data produced, in particular in the last two decades and in part reviewed here, is incorrect to say that type 2 diabetes “associates” with obesity but rather is time to acknowledge that it is caused by overweight and obesity or, in general, by an increase fat mass in the visceral depots as a sign of positive energy balance.

**Fig. 5:**
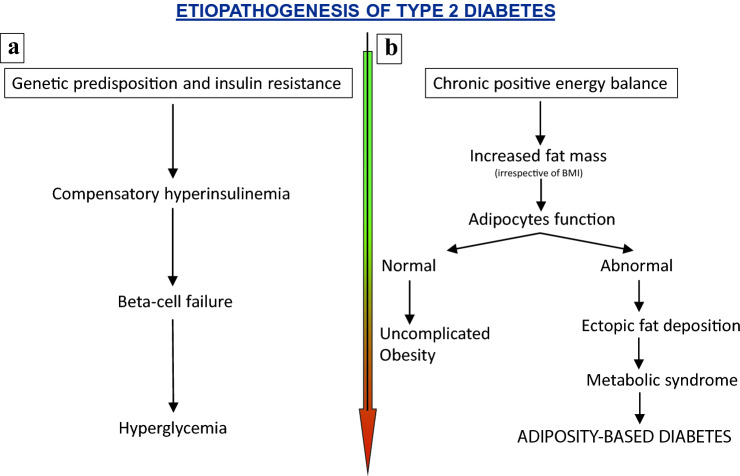
Etiopathogenesis of type 2 diabetes: the old (**a**) and new (**b**) flow charts

Therefore, it would be reasonable to move toward an etiologic definition. We recognize that it is not an easy task to identify a concise and clear etiologic definition, but we believe that this would translate in a remarkable help in better prevent, treat and, may be, reverse the disease. To say to a patient: “you have a type x disease” is the perfect way to create an initial barrier. We would like to propose the definition “adiposity-based diabetes”, although we recognize that it could be more elegant to utilize the definition that Eleazar Shafrir and Itamar Raz proposed in 2003: *diabetes lipidus* [100]. Both definitions, pointing to the energy surplus as the primum movens, would have the advantage to encompass the full spectrum of adiposopathy, irrespectively of BMI: from lipodystrophies with low BMI to severe obesity (Fig. 5).

## Have diagnostic procedure and/or pharmacologic therapy been targeted to insulin sensitivity?

The glucose clamp [36] is the gold standard for the measurement of insulin sensitivity but it is quite complex and time-consuming; many others simpler indices have been developed over the years [101] that however, although validated against the reference method, have little value in the single individual.

Today, there is no routinely and validated diagnostic methods to measure insulin sensitivity and, above all, there is no practical need to such a measurement, neither to predict type 2 diabetes nor to monitor the glycemic and metabolic control once the disease ensues.

As far as drugs affecting insulin sensitivity are concerned, metformin has been considered for many years one of such drugs; however, at the whole-body level, metformin itself does not affect insulin sensitivity in muscle or adipose tissue [102]. Instead, it has been shown to inhibit hepatic gluconeogenesis [103].

The only class of antihyperglycemic drugs that increases insulin sensitivity as principal mode of action is that of thiazolidinediones [104]. However, they enhance insulin sensitivity indirectly and fit well as drugs for the treatment of hyperglycemic conditions characterized by dysfunctional adipose tissue. Being ligands for peroxisome proliferator–activated receptor (PPAR)-gamma, a nuclear hormone receptor expressed predominantly in adipose tissue, they increase the number of small adipocytes in white adipose tissue, thus counteracting the fibro-inflammatory transformations of large adipocytes and enhancing the storage potential of the energy surplus [105].

To the best of our knowledge, there are no direct insulin sensitizers (i.e., molecules that stimulate insulin signalling) in pharmaceutical pipelines. Such drugs, in the face of continuous positive energy balance, would lead to intracellular hypermetabolism in insulin target tissues with possible deleterious effects; not mentioning the possibility of increasing the cancer risk due to proliferative and antiapoptotic effects.

Therefore, after half a century from the initial modern definition, insulin resistance did not add any clinical tool or treatment in the management of type 2 diabetes.

## Conclusions

Adiposity, sedentariness and age are the major contributors to the development of insulin resistance. In severe cases, acanthosis nigricans may develop as well as hyperandrogenism and polycystic ovary syndrome in females. Moreover, hyperinsulinemia may exert potent proliferative effects, either directly or by cross-reacting with the insulin-like growth factor 1 (IGF-1) receptor [14, 106] thus increasing the risk of cancer in obesity and type 2 diabetes.

It is not our intention to underestimate the pathophysiological role of insulin resistance, but rather to counteract the overestimation that took place in the past and that, by inertia, continue somehow nowadays. Insulin resistance became a myth and myths are hard to dispel. However, science has the duty to combat myths and dogmas; these are enemies of the scientific method and its empirism.

A huge amount of scientific evidences has been produced, part of which are presented herein, that clearly demonstrated that peripheral insulin resistance is devoid of the pivotal physiopathological role originally proposed. This concept began to be hypothesized years ago [107]. More recently, obesity was recognized as associated with increased basal and postprandial beta-cell insulin secretion even in the absence of insulin resistance [108]. In this sense, increased insulin secretion in obese subjects appears associated with excess adiposity itself and not simply a compensatory response to insulin resistance.

In summary, the data discussed in this review show that type 2 diabetes is mostly caused by a chronic condition of positive energy balance with the consequent deposition of caloric surplus in liver and pancreas when adipocytes become dysfunctional and, as long as the beta-cells are not irreversibly damaged by long-lasting disease [87], the induction of a negative energy balance is able to reverse type 2 diabetes.

In conclusion, is time to change the dogma, type 2 diabetes is not “often associated with overweight or obesity” but overweight or obesity often cause type 2 diabetes.

### What is already known on this subject?

Type 2 diabetes is defined as a complex nosographic entity caused by the interplay of insulin resistance and beta cell failure. Although increased fat deposition is always been regarded as a major risk factor, its etiological role have never been fully recognized.

### What does this study add?

To the best of our knowledge this is the first time that, within an historical perspective, data from lipodystrophies, receptoropathies, genetic studies and trials on diabetes reversibility have been put together to redraw the etiology of type 2 diabetes.

This review reappraises the role of insulin resistance in the development of type 2 diabetes and provide strong evidences that hyperglycemia ensues when caloric surplus in the form of fat, that no longer can be stored in adipose tissue, infiltrates liver and pancreas, progressively blunting insulin secretion. As long as the beta-cells are not irreversibly damaged by long-lasting disease, the induction of a negative energy balance is able to reverse type 2 diabetes. Reversibility rules out intrinsic/genetic defects pointing strongly to chronic positive energy balance being the cause.
